# Inverted Sinonasal Papilloma Masquerading as a Malignancy - Report of an Unusual Case

**DOI:** 10.7759/cureus.526

**Published:** 2016-03-09

**Authors:** Harikrishnan Prasad, Ranganath Sruthi, Krishnamurthy Anuthama, Mahendra Perumal, Ranganathan Parthasarathy

**Affiliations:** 1 Oral and Maxillofacial Pathology and Microbiology, KSR Institute of Dental Science and Research; 2 Oral and Maxillofacial Pathology, Consultant Oral Pathologist; 3 Oral and Maxillofacial Surgery, KSR Institute of Dental Science and Research; 4 Neurosurgeon, Sri Vijaya Hi-Tech Hospitals, Erode

**Keywords:** schneiderian papilloma, sinonasal papilloma, inverted papilloma, intracranial extension

## Abstract

Inverted sinonasal papilloma (ISP) is a benign epithelial neoplasm arising from the Schneiderian membrane. We report a case of ISP in a 50-year-old male that clinically presented as a polypoid mass in the nasal cavity. Imaging studies revealed it to be an aggressive lesion showing intracranial extension. On histopathological examination of the excised specimen, a diagnosis of ISP was arrived at. However, an extensive sampling of the tissue revealed no evidence of any malignant transformation. Taking into account the suggested viral aetiology for such lesions and the aggressiveness observed in this case, human papillomavirus (HPV) profiling was done but it turned out to be negative. Only one other case of inverted sinonasal papilloma arising from the nasal cavity and involving the brain has been reported in the literature to date. Considering the alarming clinical course in spite of its benign nature, it is important for the pathologist and surgeon to be well informed about this lesion.

## Introduction

Schneiderian papillomas or sinonasal papillomas are benign epithelial neoplasms arising from the Schneiderian membrane that lines the nasal cavity and paranasal sinuses. Histomorphologically, there are three types: exophytic, inverted, and oncocytic, with the inverted type accounting for about 47% of all sinonasal papillomas [1]. Inverted sinonasal papilloma (ISP) may affect individuals of any age but is more common in the fifth and sixth decades of life with a male predilection. To date, the exact etiology of ISP is not known. However, the search for a viral etiology is part of a growing trend where the role of infectious agents in the induction and neoplastic transformation of ISP is being explored. Association of human papillomavirus (HPV) Types 6 and 11 with benign ISP and Types 16 and 18 with its malignant subsets has been suggested. However, its exact role is still controversial, probably due to the disparity observed in its detection rate in ISP [2]. We report a case of aggressive ISP with intracranial extension, which, despite its behavior, turned out to be a benign tumor. To the best of our knowledge, this is the second case of ISP arising from the lateral nasal wall with extension into the frontal lobe of the brain being reported in the English-language medical literature.

## Case presentation

**Figure 1:**
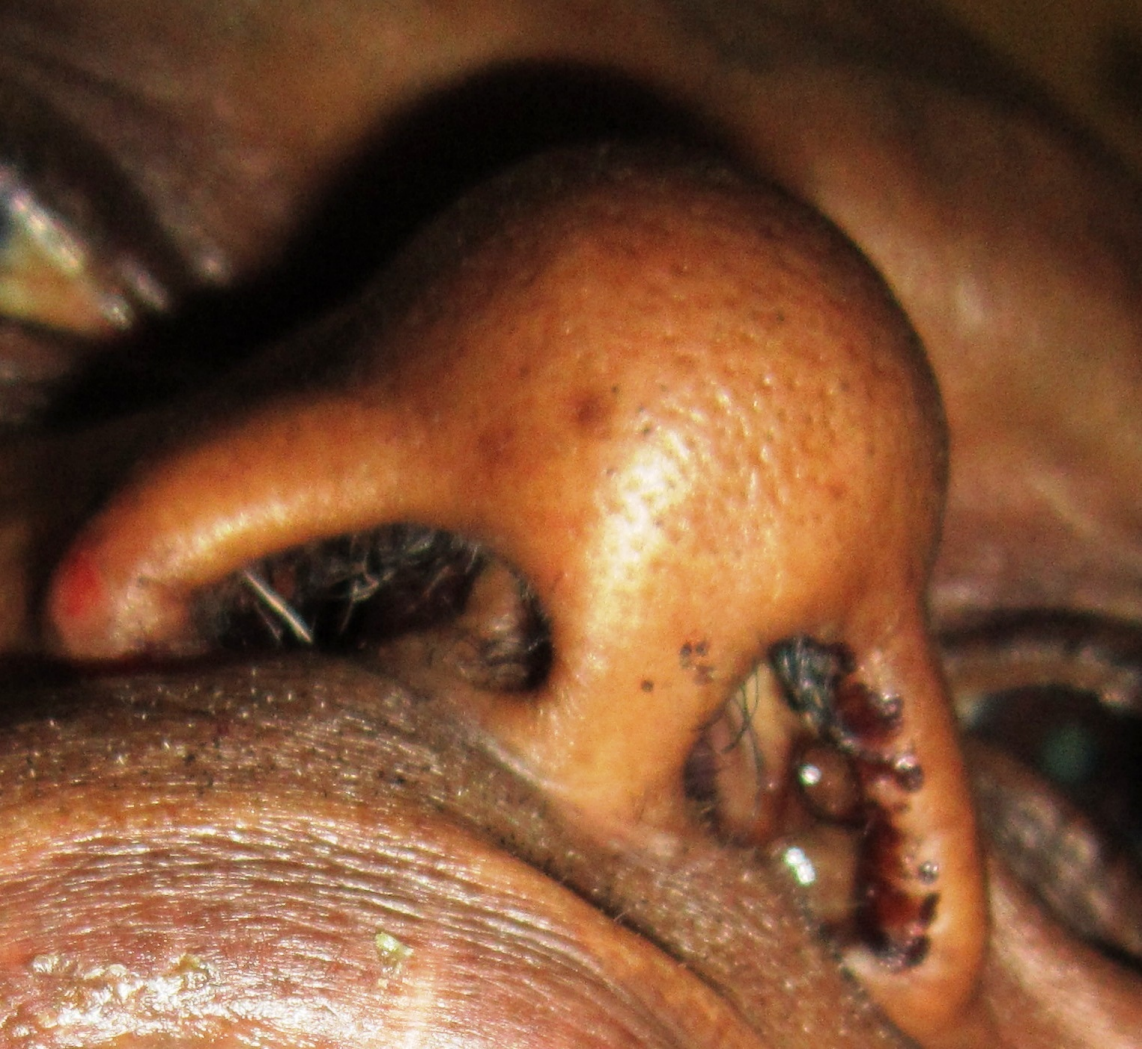
Polypoid mass seen filling the left nasal cavity

A 50-year-old male patient presented with complaints of nasal stuffiness and bilateral purulent blood-tinged nasal discharge of eight years duration. He experienced vomiting, occasional loss of consciousness, anosmia, and headache for the last one month. The patient also gave a history of undergoing an operative procedure for removal of a nasal growth 10 years ago. He had been under Siddha treatment (traditional Indian medicine) for the same problem since that time. On physical examination, a diffuse swelling was seen on the left side of the nose, obliterating the nasal fold and causing flaring of the left ala. A reddish pink polypoid mass partially filling the left nasal cavity, with associated purulent bloody discharge, was noticed (Figure 1). On palpation, it was friable, tender, and readily bled on provocation. Informed patient consent was obtained for treatment. No reference to the patient's identity is present in this paper.

**Figure 2:**
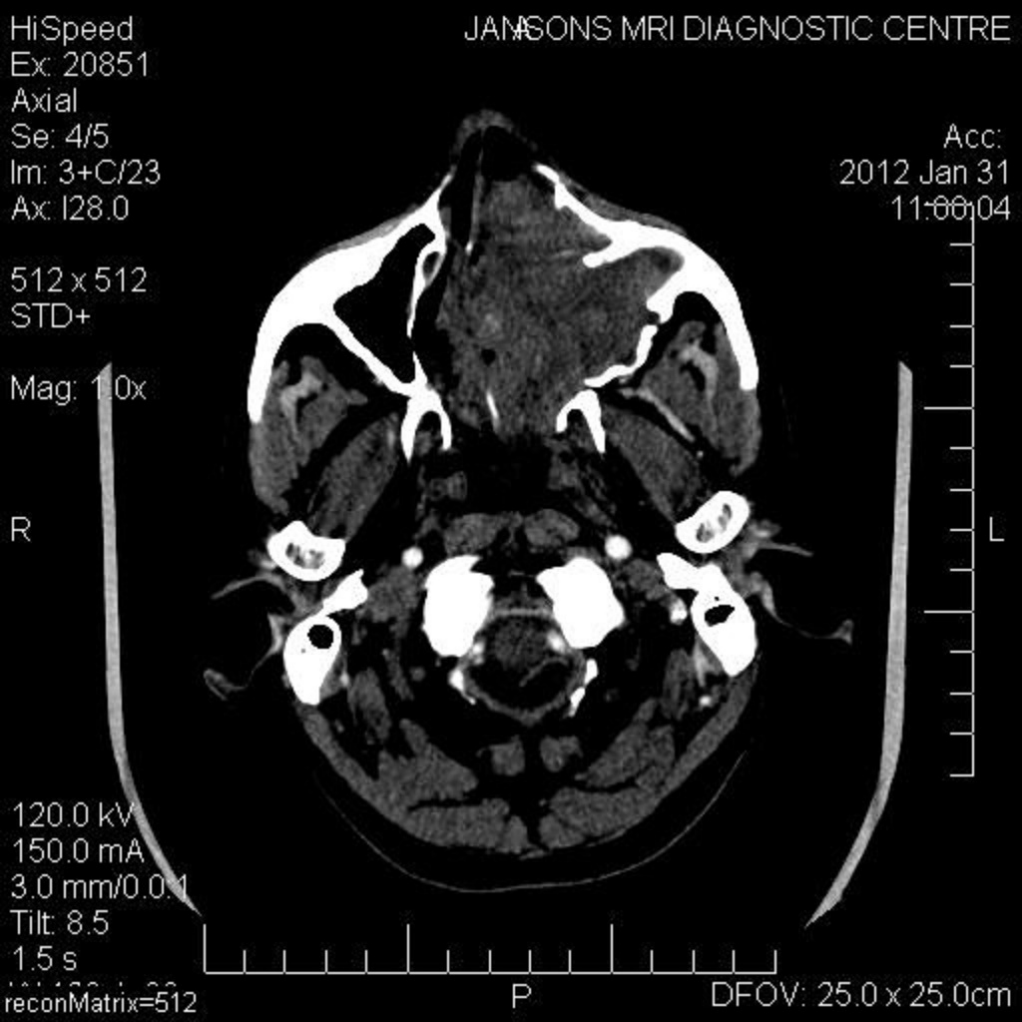
CT showing a large mass occupying the entire left nasal cavity and maxillary antrum, and also extending towards the right nasal cavity

**Figure 3:**
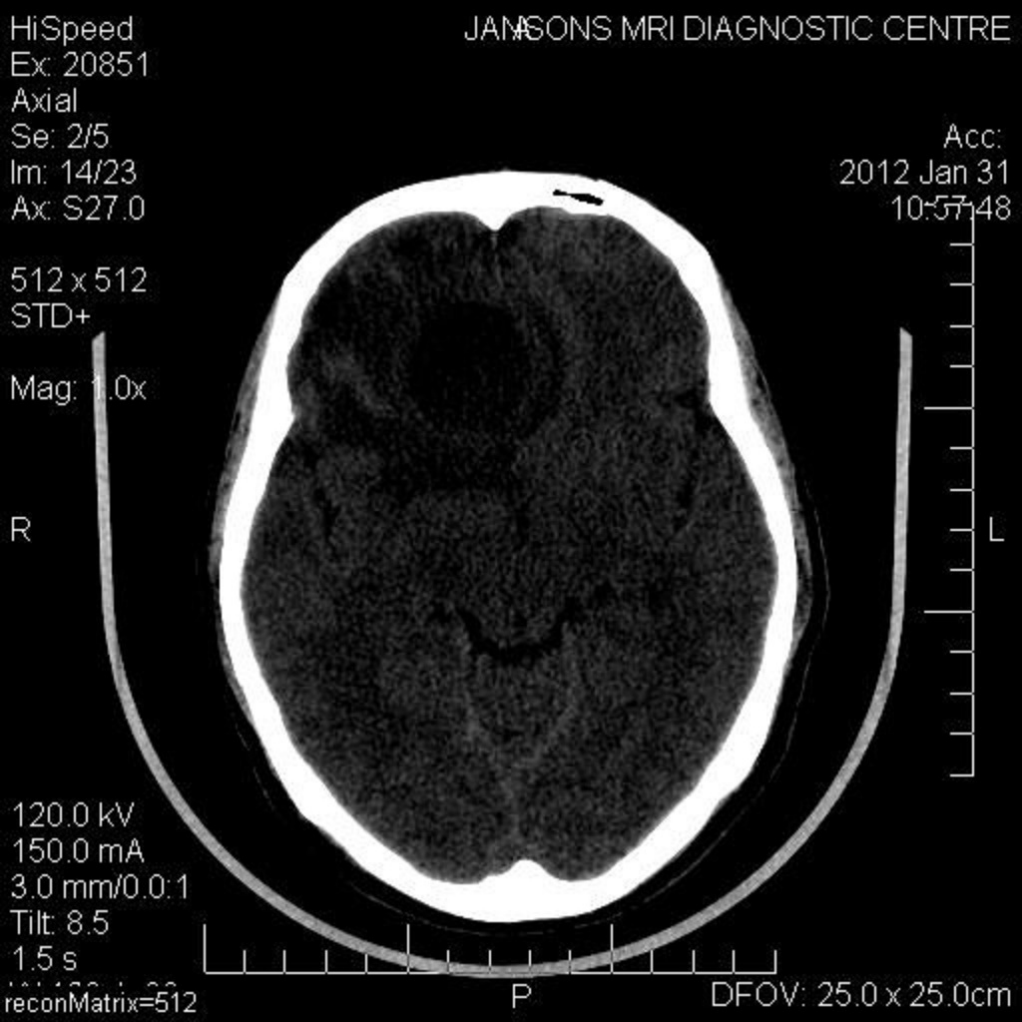
Space-occupying lesion noticed in the right frontal lobe of brain

A computed tomographic (CT) scan of the head and neck with contrast revealed an enhancing soft tissue mass arising from the left lateral nasal wall, filling the left maxillary sinus, and partially extending into the right nasal cavity (Figure 2). It further extended into the anterior cranial fossa by perforating the roof of the nasal cavity and the posterior table of the frontal sinus, forming a cystic space-occupying lesion in the right frontal lobe of the brain (Figure 3). The presence of a bony window revealed the involvement of the cribriform plate of the ethmoid and close proximity to the pterygoid plates.

**Figure 4:**
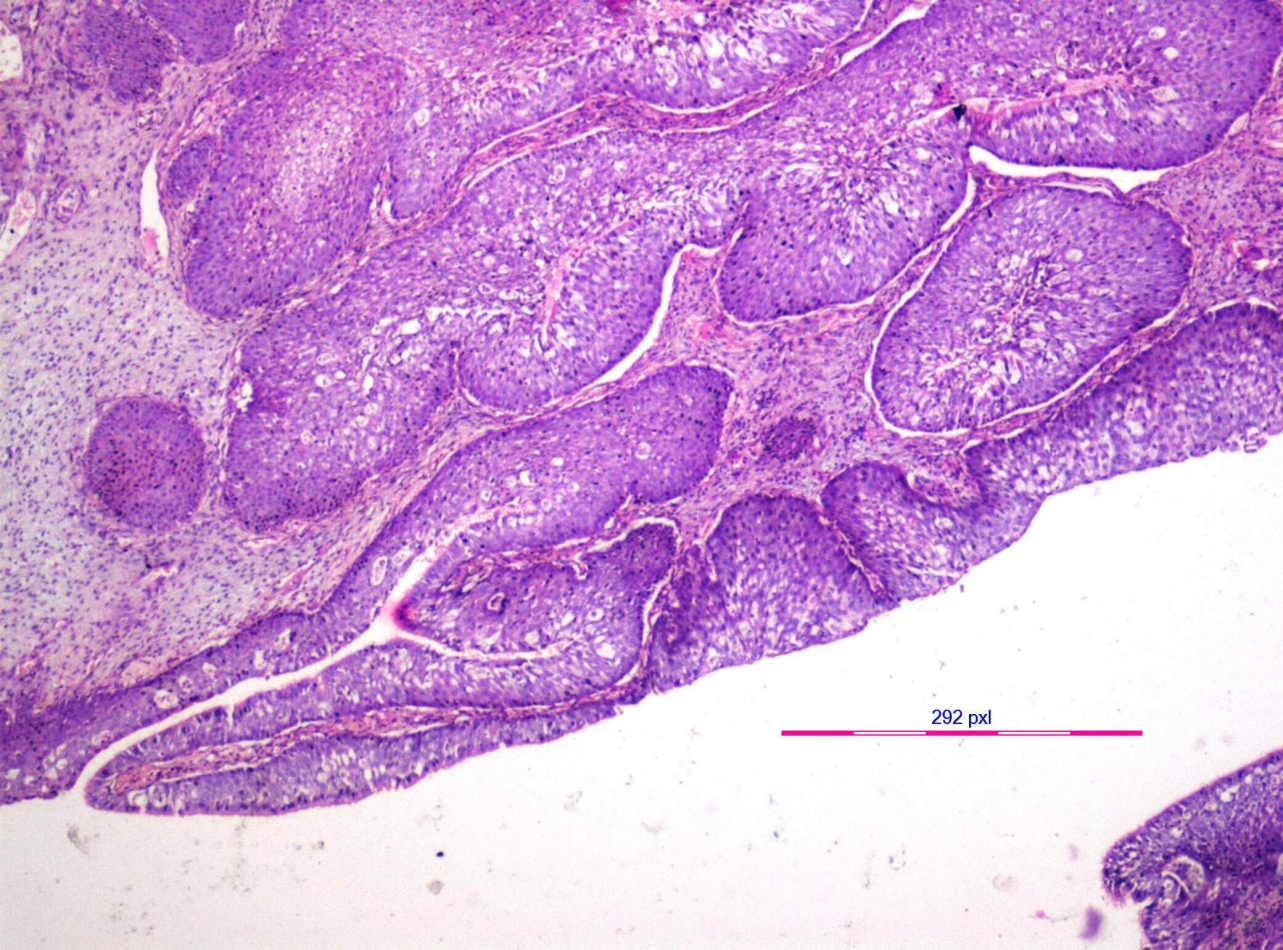
Endophytic proliferation of stratified squamous non-keratinized epithelium noticed in the incisional biopsy. (Hematoxylin & eosin stain x100 magnification)

**Figure 5:**
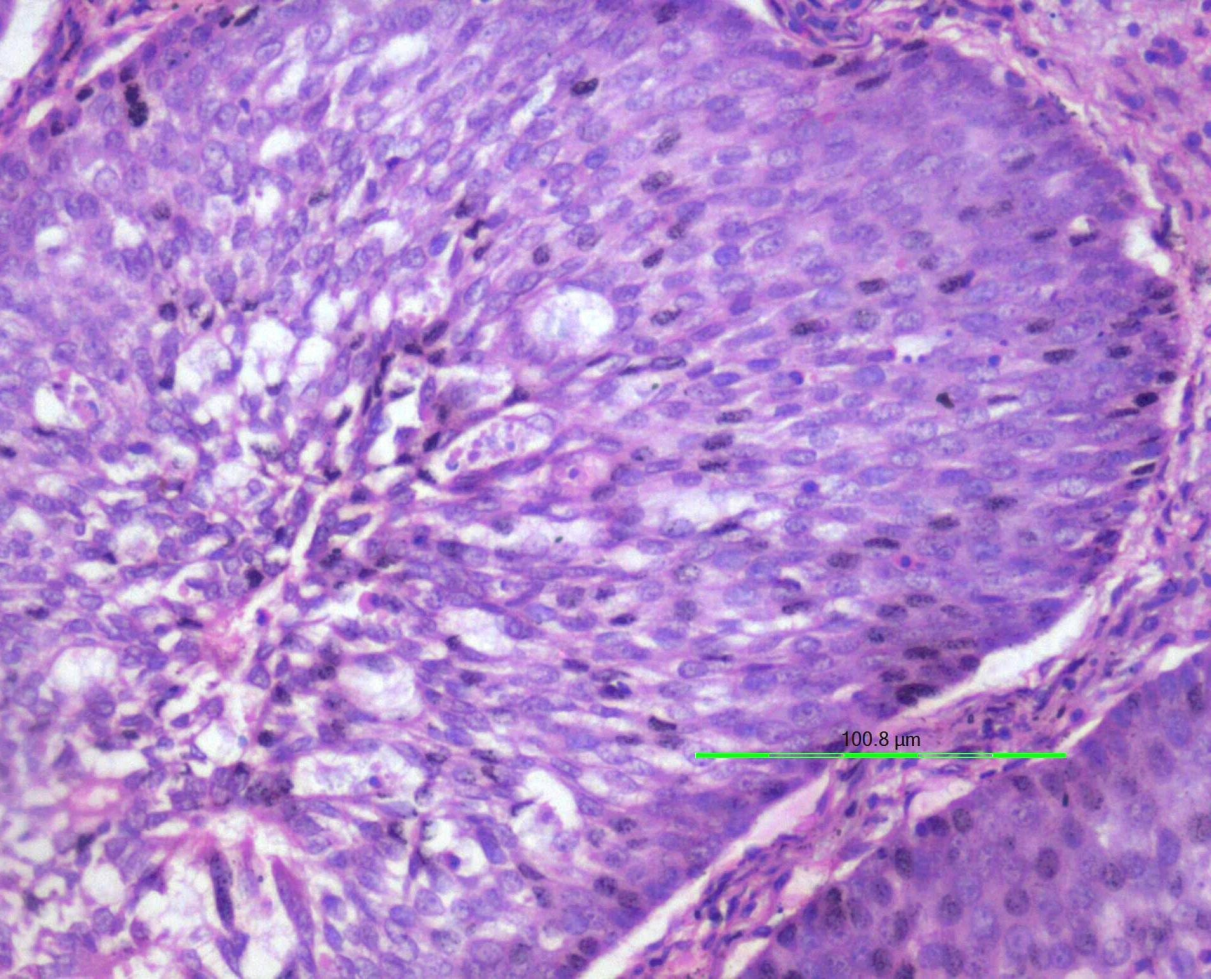
Epithelium showing koilocytic change and very few mitosis. (Hematoxylin & eosin stain x200 magnification)

An incisional biopsy of the tissue from the nasal region on histopathological examination revealed an extensive proliferation of thick, non-keratinizing squamous epithelium showing endophytic growth in most of the areas (Figure 4). Goblet cells and microcysts filled with neutrophils were seen within the epithelium. A few cells showed koilocytic change (Figure 5). Respiratory-type epithelium was also evident in several areas. However, the basement membrane was intact, although mild atypia was noticed in certain areas. The mitotic index was unremarkable. Based on these findings, a diagnosis of ISP was made.

**Figure 6:**
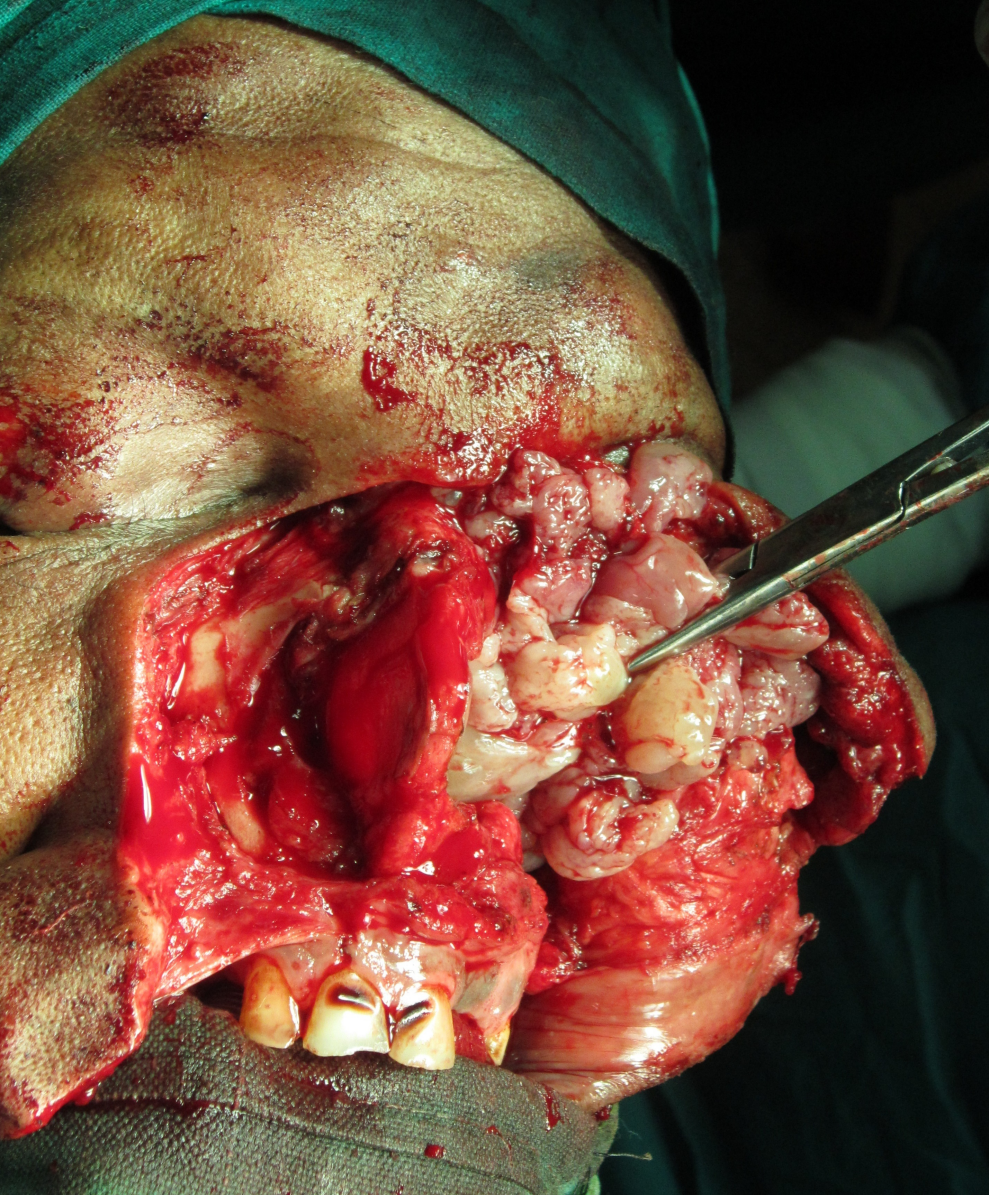
Surgical removal of intranasal lesion showing gross polypoid appearance

**Figure 7:**
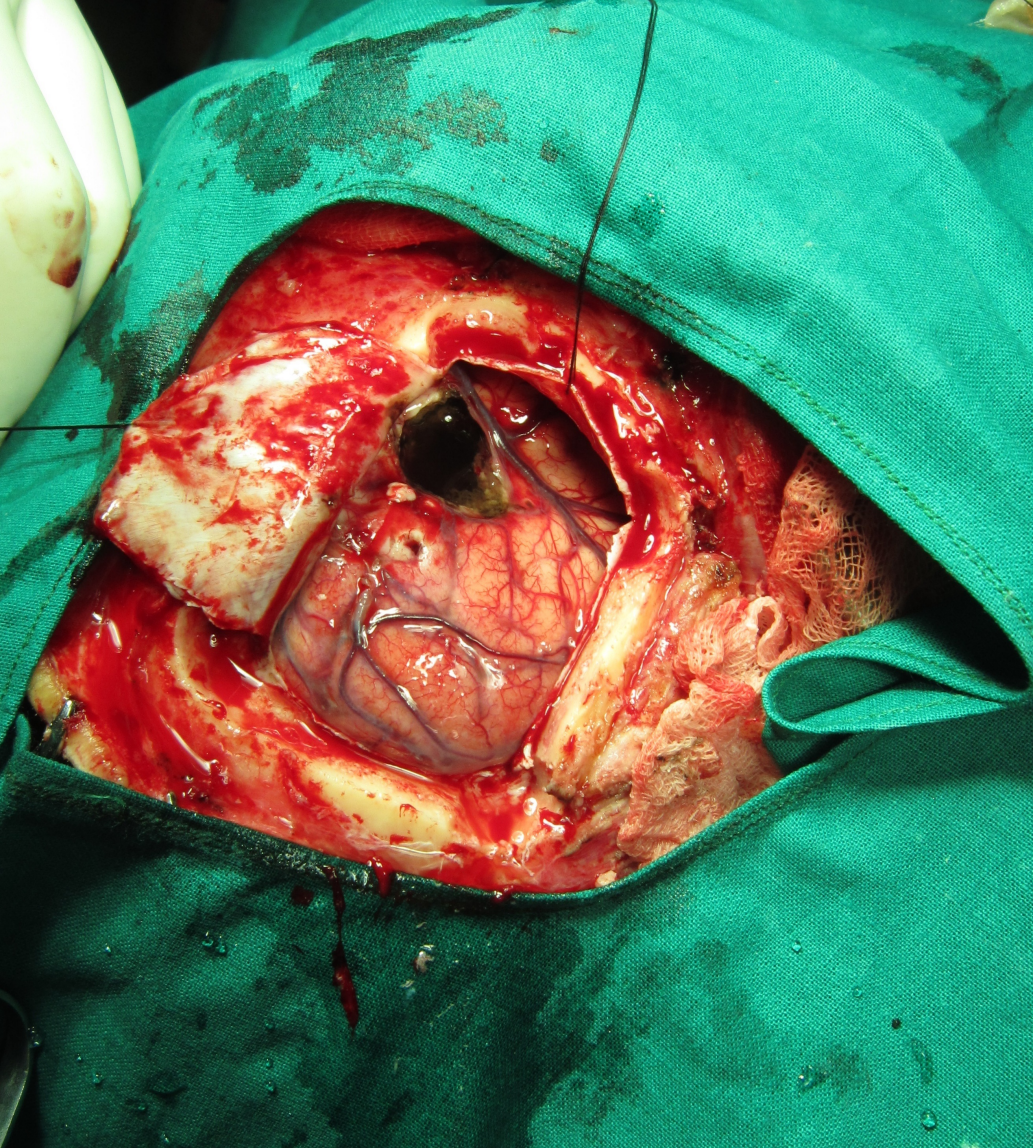
Bifrontal craniotomy approach to remove the lesion in the frontal lobe

Following this, a wide excision of the lesion was planned. A bifrontal craniotomy was performed. A complete removal of the cranially extending polypoidal sinonasal growth was carried out by an open approach (Figures 6-7). Postoperative recovery was uneventful. The histopathological evaluation of the excised tissue from the sinonasal region was consistent with the incisional biopsy report, and the diagnosis of ISP was confirmed. Immunohistochemical analysis of the tissue from the nasal region was negative for HPV 16 and 18. On neuropathological examination at the National Institute of Mental Health and Neuro Sciences, Bangalore, the excised cranial bits showed dense lymphoplasmacytic and histiocytic infiltration of the brain parenchyma, suggestive of a pyogenic abscess formation with gliosis.

## Discussion

ISP is a benign lesion with a prevalence of 0.5 – 4% of all sinonasal tumors [3]. Califano, et al., through their molecular genetics investigation, showed that ISP was a true neoplasm arising from single progenitor cells [4]. There are two hypotheses proposed for the origin of ISP - metaplastic inversion of the surface epithelium or metaplasia of surface epithelium and ductal epithelium [3]. Although benign, ISPs are often locally aggressive. Sometimes the invasion is so extensive that the adjacent vital structures may be compromised to the extent that it may even cause death. This feature, together with its propensity for recurrence and malignization, makes the diagnosis and management truly challenging [5].

Although many factors, such as allergy, chronic sinusitis, human papillomavirus (HPV), Epstein-Barr virus (EBV), environmental carcinogens, and tobacco smoking, have been proposed, the actual etiology of ISP is still unknown. Documented reports on the association of ISP with EBV and HPV are variable [1, 6]. Ever since Brandsma, et al. demonstrated the presence of HPV DNA in ISP, an evaluation of viral etiology has been the focus of several studies [7]. About 15% – 35% of head and neck cancers are known to be associated with HPV infection [1]. HPV is suggested to be one of the causative factors in the pathogenesis of ISP, although it may not be essential for the induction of the tumor. HPV Subtypes 6 and 11 are said to be associated with benign ISP while Subtypes 16 and 18 are associated with the malignant counterpart [8]. Therefore, it has been suggested that HPV subtyping enables delineation of the benign and malignant clinical course of ISP while also predicting the probability of recurrence [1].

ISPs predominantly occur in males with a peak incidence in the fifth and sixth decades of life [1]. According to Batsakis, et al., patients with ISP gave a history of nasal polyposis with repeated polypectomy [9]. ISPs present as unilateral polyps. Symptoms in ISP develop only when either the sinus is obstructed by the tumor or when the tumor extends beyond the confines of the sinus. Thus, early neoplasms are usually asymptomatic. When symptoms do occur, they are non-specific. Typical complaints include nasal obstruction and sinusitis. However, ISP invading adjacent structures produces atypical symptoms secondary to the invasion, like anosmia, proptosis, frontal headache, and blurring of vision, depending upon the site of extension [1].

Early diagnosis of ISP is necessary for optimum management. Clinical examination using nasal endoscopy may facilitate the assessment of the local extent of the tumor while also allowing tissue sampling for biopsy. However, aggressive ISPs located within the craniofacial skeleton cannot be accurately assessed using nasal endoscopes. Assessment of such cases can be achieved with the help of computed tomography (CT) scans and magnetic resonance imaging (MRI).

Differential diagnosis of ISP includes antral choanal polyps, allergic fungal rhinitis, giant cell granuloma, and mucocele. A diagnosis of ISP just based on clinical and radiographic features without the histopathology report cannot be arrived at, as it lacks pathognomonic features [2]. The World Health Organization classified sinonasal papilloma into three histological types: exophytic, inverted, and oncocytic [10]. The distinctive microscopic findings of ISP include an endophytic or inverted growth of multilayered epithelium into the underlying stroma with intact basement membrane and minimal nuclear atypia. The epithelium is non-keratinizing squamous, transitional, or respiratory type, admixed with mucocytes and microcysts. Stroma ranges from dense fibrous to loose myxoid, with mixed inflammation [1]. Histopathology of our case revealed a similar picture. According to Pitak-Arnnop, et al., despite its benign nature, ISP exhibits three characteristic features - local aggressiveness, propensity for recurrence, and association with malignancy [2]. Seventy-five percent of patients with ISP have varying degrees of bone destruction in the form of thinning, remodeling, erosion, or sclerotic bone changes. Bone erosion in ISP is often due to pressure necrosis. Such ISPs may invade sites like the orbit, cranial cavity, or middle ear cavity. Therefore, the underlying vital structures may be involved by the tumor at a very early stage [1, 11].

Mirza, et al. suggest that intracranial extension is rare unless malignant transformation is present [12]. However, Tomazic, et al. were of the opinion that intracranial expansion by ISP is possible, despite its histology showing no signs of malignancy [5]. In our case, the lesion extended cranially after eroding the roof of the nasal cavity and cribriform plate of the ethmoid into the anterior cranial fossa and caused degeneration of the frontal lobe brain parenchyma. Such marked extension might be observed in cases which undergo a broad malignant transformation. However, even after extensive sampling of the tissue, we did not find foci of malignancy. To the best of our knowledge, based on our search using the PubMed search engine with the keywords ‘benign’, ‘inverted papilloma’, ‘anterior cranial fossa’, and ‘HPV’, this appears to be the second report of a benign ISP arising from the lateral nasal wall and extending into the frontal lobe of the brain. The only other case was an aggressive ISP that expanded intracranially, infiltrating the frontal lobe, as reported by Tomazic, et al. in 2011.

The association of ISP with malignancy may be manifested either as synchronous carcinoma or as metachronous carcinoma, with squamous cell carcinoma (SCC) being the most predominantly associated malignancy. The frequency of carcinoma associated with ISP is reported to be 1 – 50% [1]. The mean duration of carcinoma to develop from ISP was 52 months, according to Mirza, et al. [12]; however, as short as a three months span was reported by Tomazic, et al. [5]. There are many reports of a change from a primarily benign ISP to carcinoma in situ and to invasive carcinoma. The actual cause that triggers malignancy in ISP is not known yet [1]. There is some evidence that the presence of HPV Subtypes 16 and 18 was related to malignization of ISP and in the pathogenesis of squamous cell carcinoma originating from the nasal cavity and paranasal sinuses. A significant association was also found between identification of HPV DNA in ISP and recurrence after surgical excision [13]. Progression of ISP to dysplasia and malignancy may result from (a) secondary infection of HPV 16/18, (b) loss of tumor suppressor gene, (c) integration of HPV 16/18 and HPV 6/11, or (d) a combination of the above three factors [2]. Immunotyping with p21, p53, Ki67, proliferating cell nuclear antigen (PCNA), epidermal growth factor receptor (EGFR), and transforming growth factor alpha (TGF - α) are predictive of malignant transformation [1-2]. Eggers, et al. mention that no reliable coherence has been established between such histopathologic parameters and the possibility of recurrence or malignancy association in a diagnosed ISP. However, observation of these parameters might help to identify cases with a high risk for metachronous SCC [1, 14].

Lawson, et al. highlighted the likelihood of recurrence in ISP where HPV detection is positive [8]. According to Califano, et al., there is every possibility of a malignancy in a recurrent ISP arising after the excision of a benign ISP [4]. Thus, HPV infection may be an early event in a multistep process of malignant transformation of an ISP [8]. The locally aggressive behavior and history of nasal growth excision, evidenced in our case, instigated us to rule out the possibility for a malignant transformation from a secondary infection by a high-risk HPV. With this notion, HPV 16 and 18 profiling were done, which turned out to be negative. During a 20 month follow-up, except for mild nasal discomfort, the patient was asymptomatic.

The treatment of choice for ISP is a complete surgical excision using either an open or endoscopic approach. Staging allows the assessment of outcome following different approaches. Our case falls into Stage IV of the Krouse classification [15] and Group C of the Cannady, et al. staging system [16]. Surgical intervention of IP with intracranial involvement is truly challenging. In such cases, complete excision of the lesion is technically difficult owing to the anatomic location [17]. Recurrence in ISP tends to be variable. About 6 – 75% of the cases reported had a recurrence after surgical removal. Recurrence was related to several factors, such as HPV infection, young age, smoking, and increased mitotic index. However, most recurrences reported were due to incomplete resection. The type of surgical procedure opted (open or endoscopic approach) did not contribute any difference to the recurrence rate [1]. Histopathologic evaluation of the bone underlying ISP showed numerous irregular surfaces, which can hinder complete tumor excision as microscopic nests of the tumor may be hidden within such crevices. It is suggested that ISPs that recur after surgical treatment may represent a subset of lesions with an inherent aggressiveness [1-2, 8]. Though tumor size, viral infection, stage and grade of the tumor, and modality of surgery performed are suggested to be prognostic indicators, in essence, there is no reliable parameter that can actually predict the prognosis. The propensity for delayed recurrences and incidence of malignant transformation mandates careful long-term follow-up [1].

## Conclusions

This case report highlights the deceptive behavior of inverted sinonasal papilloma. It is rare and unusual to encounter a sinonasal papilloma presenting as a space-occupying lesion in the brain and causing a major catastrophe. Although a benign lesion, local aggressiveness and a propensity for recurrence and malignant change typically differentiates inverted papilloma from the rest of the neoplasms in the sinonasal region and poses a real challenge to clinicians and surgeons. Association of HPV and ISP truly dictates the necessity for its profiling in identifying patients at risk for recurrence and to rule out malignancy.
